# Drivers of Centipede and Spider Diversity and Biomass Along an Elevation Gradient on Changbai Mountain, China

**DOI:** 10.1002/ece3.72074

**Published:** 2025-09-25

**Authors:** Zhuoma Wan, Yunga Wu, Peng Zhang, Zhijing Xie, Donghui Wu, Stefan Scheu

**Affiliations:** ^1^ School of Environment Northeast Normal University Changchun China; ^2^ Collage of Life Science and Technology Inner Mongolia Normal University Hohhot China; ^3^ Key Laboratory of Soil Resource Sustainable Utilization for Commodity Grain Bases of Jilin Province, College of Resource and Environmental Science Jilin Agricultural University Changchun China; ^4^ Key Laboratory of Wetland Ecology and Environment, Institute of Northeast Geography and Agroecology Chinese Academy of Sciences Changchun China; ^5^ State Environmental Protection Key Laboratory of Wetland Ecology and Vegetation Restoration, School of Environment Northeast Normal University Changchun China; ^6^ Jilin Provincial Key Laboratory of Animal Resource Conservation and Utilization Northeast Normal University Changchun China; ^7^ J.F. Blumenbach Institute of Zoology and Anthropology University of Göttingen Göttingen Germany; ^8^ Centre of Biodiversity and Sustainable Land Use University of Göttingen Göttingen Germany

**Keywords:** Araneidae, arthropods, biodiversity pattern, Chilopoda, predator, soil fauna

## Abstract

In the context of global change, mountain ecosystems are facing more threats than ever. Therefore, understanding spatial distribution patterns of diversity and their driving factors on mountains is gaining increasing attention. Although comprising an essential component of terrestrial ecosystems, the structure of soil fauna communities in mountain ecosystems and their driving factors have been little studied. Changbai Mountain harbors one of the most well‐preserved forest ecosystems in the temperate zone. Its high biodiversity provides an ideal setting for investigating biodiversity patterns along elevation gradients. We investigated the diversity, biomass, and community composition of two key soil predator taxa—centipedes and spiders—across eight elevations ranging from 800 to 1850 m a.s.l. Furthermore, we explored correlations between community characteristics and environmental factors. A total of 26 centipede species were identified among 2796 individuals, while 76 spider species were recorded from 2327 individuals. Both centipede and spider richness, biomass, as well as spider density, decreased with increasing elevation. Climatic variables and litter quality were identified as the primary drivers influencing the richness, biomass, and community composition of both taxa. Specifically, changes in temperature and precipitation associated with elevation were identified as the main drivers of changes in diversity, biomass, and community composition. Litter quality, including litter pH, total phosphorus, total carbon, total nitrogen, and N/P ratio, was of secondary importance. Overall, the results provide critical insights into the vulnerability of soil fauna to global climate change and highlight the need for conservation strategies that account for the complex interactions between biodiversity and environmental change.

## Graphical abstract

Changes in density, diversity, and biomass with elevation differ between centipedes and spiders. Compared to centipedes, spiders are more strongly affected by environmental changes. Climatic variables act as the main drivers of centipede and spider density, diversity, and biomass, with local habitat–related factors being of secondary importance.

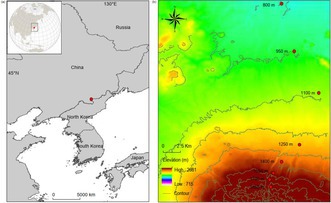

## 1 Introduction

The distribution patterns and drivers of biodiversity along geographical gradients are central topics in ecology and biogeography, and this applies to both latitudinal and altitudinal gradients (Crowther et al. 2019; Fenton et al. 2023; Quintero and Jetz 2018). Mountain ecosystems provide ideal natural laboratories for investigating these patterns as temperature, precipitation, and edaphic properties (e.g., litter and soil characteristics) change over short spatial scales and drive the assembly and characteristics of communities (Antonelli et al. 2018; Cancino‐López et al. 2022; Malhi et al. 2010; Perrigo et al. 2020). Biodiversity, density, and biomass may respond differently to these gradients, allowing deeper insight into the structuring forces of community characteristics. For instance, tropical montane forests often exhibit a mid‐elevation peak in plant diversity, while aboveground biomass typically declines with elevation due to thermal constraints on growth and enhanced carbon allocation to roots and soil (Castillo‐Figueroa 2021; Fadrique et al. 2018; Moser et al. 2011). However, the mechanisms underlying these patterns, and whether they are consistent across taxa (e.g., plants, animals, microbes) and ecosystems, remain poorly known.

Soils, which harbor approximately 59% of all species on Earth (Anthony et al. 2023), support diverse faunal communities that are essential for ecosystem processes such as decomposition, nutrient cycling, and soil formation (Decaëns et al. 2006; Coleman et al. 2017). The diversity and biomass distribution of these communities have profound ecological implications (Bardgett and van der Putten 2014; Frouz 2018; Orgiazzi et al. 2016). Recent studies on key soil invertebrate groups, such as oribatid mites (Mumladze et al. 2015) and collembolans (Xie, Sun, et al. 2022), revealed divergent elevation trends in species richness. While some taxa exhibit a hump‐shaped pattern with mid‐elevation diversity peaks, others showed a monotonic decline in diversity with increasing elevation (Khatiwada et al. 2019; Li et al. 2003; Patterson et al. 1998; Worthy et al. 2019). These contrasting patterns likely reflect taxon‐specific responses to environmental constraints, such as thermal limits, resource availability, and habitat heterogeneity. In contrast to species richness, soil faunal biomass more uniformly follows a hump‐shaped distribution with elevation due to lower temperature, reduced precipitation, and limited organic matter input at higher elevations (Leakey and Proctor 1987; Sundqvist et al. 2013; Werenkraut and Ruggiero 2014). However, the relative importance of these drivers remains debated. Despite their ecological significance, integrated studies examining both diversity and biomass patterns along elevation gradients remain scarce, limiting our ability to clarify the mechanisms responsible for major soil community characteristics, which is particularly important for their maintenance in the face of global climate change.

Centipedes and spiders, as prominent components of arthropod predators in virtually any soil, are widely distributed (Clarke and Grant 1968; Eitzinger et al. 2018; Evans et al. 2005; Mona and Atlam 2022) and play an important role in ecosystem functioning by regulating the density of prey populations (Liu et al. 2016; Samu et al. 2021; Schneider and Brose 2013). Both centipedes and spiders have been shown to sensitively respond to climatic and habitat‐related changes (Klarner et al. 2017; Samu et al. 2023). Temperature is among the most important factors in determining the development of centipedes and spiders, and, consequently, their distribution range (Cortez‐Roldán and Valdez‐Mondragón 2024; Georgopoulou et al. 2016; Moen et al. 2022; Vedel et al. 2010). Soil moisture has been found to enhance the diversity of both centipedes and spiders (Kajak et al. 2000; Poloczek 2016). However, centipedes prefer dark and humid environments to spiders (Lazorík and Kula 2015; Malumbres‐Olarte et al. 2018; Negi et al. 2025). Furthermore, habitat‐related factors, such as litter characteristics and pH, significantly affect the density and spatial distribution of centipedes and spiders in soil (Klarner et al. 2017; Langraf et al. 2023). Spiders are predominantly active in the aboveground litter layer and vegetation and tend to be more mobile and responsive to environmental variation than centipedes, particularly in temperature and vegetation structure (Ballini 2009; Foord et al. 2002; Ziesche and Roth 2013). By contrast, centipedes are primarily soil‐dwelling and often rely on buffered microhabitats in soil, which may reduce their sensitivity to broad‐scale elevational gradients (Voigtländer 2013). Therefore, we hypothesized that spider diversity, density, and biomass would show stronger elevational responses compared to those of centipedes.

Changbai Mountain, located in northeastern China, is well known for its rich biodiversity and recognized as one of the world's 36 biodiversity hotspots (Mittermeier et al. 2004; Weinzettel et al. 2018). The vegetation along the northern slopes of Changbai Mountain exhibits a distinct vertical zonation pattern (Bai et al. 2011), making it an ideal location for studying ecological patterns along elevation gradients. Previous studies have focused on the distribution pattern of small soil detritivore animal taxa across different elevation and vegetation zones, including Oribatida (Liu et al. 2023a, 2023b) and Collembola (Sun et al. 2020; Xie, Sun, et al. 2022), as well as ground beetles (Ji et al. 2024) and bacteria (Han et al. 2018). However, the elevation patterns observed in these taxa were inconsistent and call for further investigations in particular on other trophic groups such as predatory soil arthropod taxa. To address this knowledge gap, we focused on centipedes and spiders, aiming to explore their changes in diversity and biomass with elevation and identify the factors responsible for these changes. We hypothesized that (1) density, diversity, and biomass of centipedes and spiders decrease with increasing elevation, with this being more pronounced in spiders than in centipedes, and (2) temperature and precipitation are the major drivers of both centipede and spider density, diversity, biomass, and community composition, but their relative importance differs and depends on litter characteristics.

## 2 Materials and Methods

### 2.1 Study Area

The study was conducted in the Changbaishan Nature Reserve (41°41′49″–42°25′18″ N, 127°42′55″–128°16′48″ E), Jilin Province, northeast China. The reserve comprises some of the best protected, fully developed temperate forests in Asia (Stone 2006; Xue and Tisdell 2001). The area is characterized by a typical temperature continental monsoon climate (Zhang et al. 2015), with pronounced changes in temperature and precipitation with increasing elevation. Changbai Mountain ranges between 600 and 2745 m, and across the elevation gradient annual mean precipitation ranges between 683 and 955 mm (Xie, Chen, et al. 2022) and annual mean temperature between −4.17°C and 2.71°C. Along the elevation gradient, Changbai Mountain is characterized by five distinct vegetation types exhibiting clear elevation zonation, including coniferous and broad‐leaved mixed forest below 1100 m, mixed coniferous forest between 1100 and 1500 m, subalpine coniferous forest between 1500 and 1800 m, birch forest between 1800 and 2100 m, and alpine grassland above 2100 m (Bai et al. 2011). For more details on the study sites, see Table 1 (Zhang et al. 2024).

**TABLE 1:** Summary of the vegetation types and dominant plant species of the sampling sites along the elevational gradient on Changbai Mountain.

Elevation (m)	Vegetation types	MAT (°C)	MAP (mm)	Soil types	Dominant plant species
800	Mixed coniferous and broad‐leaved forests	2.71	683	Albi‐Boric Argosols	*Pinus koraiensis* , *Acer mono*,*Tilia Amurensi*, *Ulmus propinoua*, *Quercus Mongolia*
950	Mixed coniferous and broad‐leaved forests	1.82	764	Albi‐Boric Argosols	*Pinus koraiensis* , *Acer mono*,*Tilia Amurensi*, *Ulmus propinoua*, *Quercus Mongolia*
1100	Mixed coniferous forests	1.58	762	Albi‐Boric Argosols	*Pinus koraiensis* , *Picea jezoensis*, *Abies nephrolepis*, *Larix olgensis*
1250	Mixed coniferous forests	0.36	782	Bori‐Udic Cambosols	*P. koraiensis* , *Picea jezoensis*, *Abies nephrolepis*, *Larix olgensis*
1400	Mixed coniferous forests	−0.61	809	Bori‐Udic Cambosols	*Pinus koraiensis* , *Picea jezoensis*, *Abies nephrolepis*, *Larix olgensis*
1550	Subalpine mixed coniferous forests	−1.11	835	Umbri‐Gelic Cambosols	*Picea jezoensis*, *Larix olgensis*, *Abies nephrolepis*
1700	Subalpine mixed coniferous forests	−1.72	880	Umbri‐Gelic Cambosols	*Picea jezoensis*, *Larix olgensis*, *Abies nephrolepis*
1850	Birch forests	−2.83	902	Umbri‐Gelic Cambosols	*Betula ermanii*, *Rhododendron aureum*

### 2.2 Sampling

**FIGURE 1:**
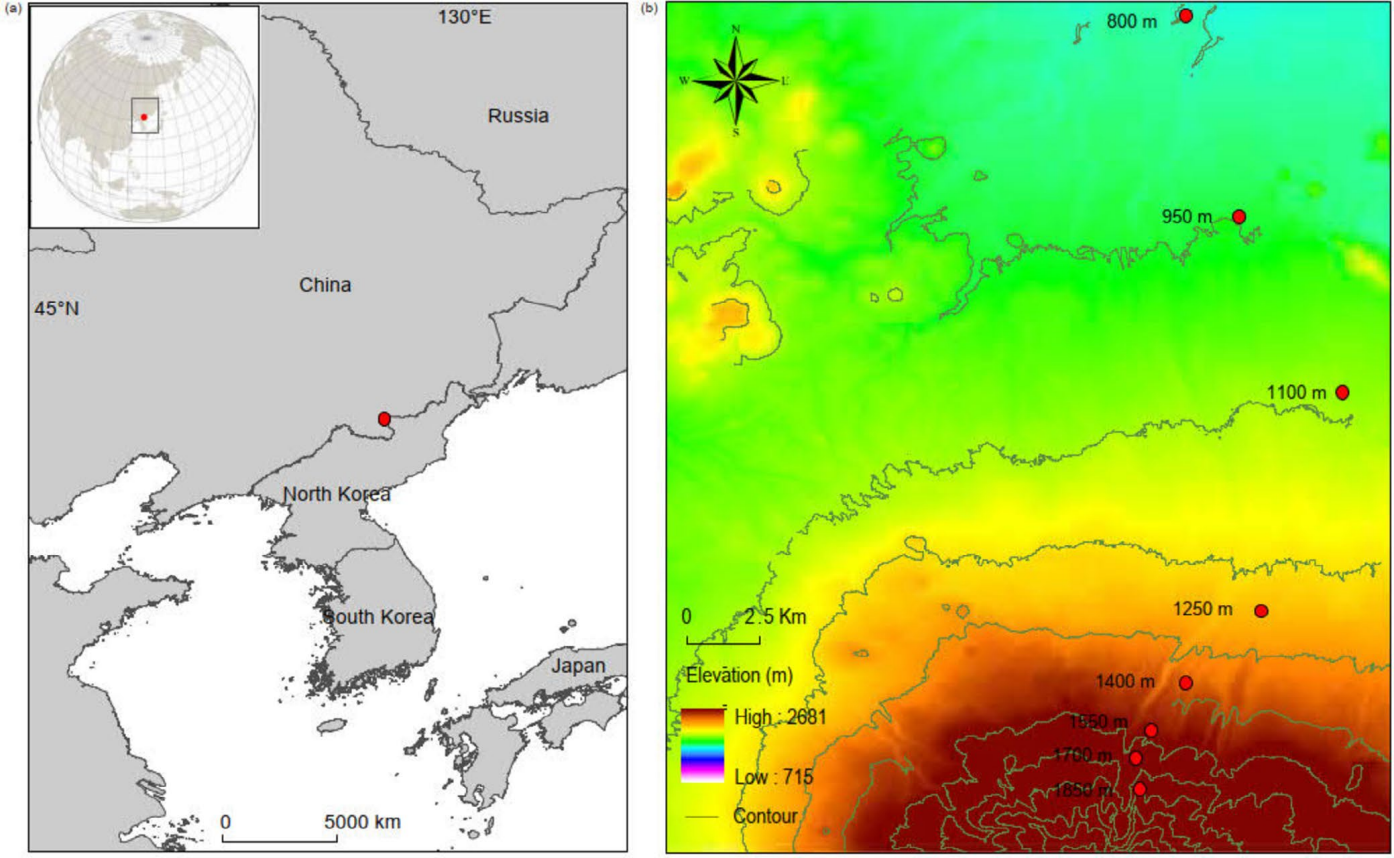
Location of the study area on Changbai Mountain, northeast China (red dot) (a) and sampling locations along an elevation gradient from 800 to 1850 m (b).

The study sites were located on the northern slope of Changbai Mountain. Eight forest sites, spanning from 800 to 1850 m, were selected at intervals of approximately 150 m (Figure 1). Samples were taken in 2021 between September 15 and 22, resulting in a total of 64 samples (8 elevational sites × 8 plots). To minimize spatial autocorrelation of centipedes and spiders, eight 5 m^2^ plots were randomly selected at each site, with each plot positioned > 100 m apart (Keitt et al. 2002). At each elevational site, a team of eight persons conducted hand‐sorting for centipedes and spiders, spending approximately 1.25 h per 5 m^2^ plot. The procedure involved screening leaf litter and the top 5 cm of soil from a 1 m^2^ subarea, sifting litter through a 1 cm mesh sieve, and manually collecting individuals. All eight plots at each elevation site were completed within 1 day. Centipedes and spiders were collected by hand and transferred into 20 mL tubes containing 70% ethanol, and then transferred to the laboratory and stored at −20°C. Material from the mineral soil was placed successively in white trays and carefully inspected by hand; centipedes and spiders were collected as described above. Additionally, five samples of litter material were taken from each plot using a corer of 5.5 cm in diameter and thoroughly mixed, and the material was used for analyzing pH, total carbon, nitrogen, and phosphorus.

### 2.3 Species Identification

Centipedes and spiders were identified to morphological species under a stereomicroscope (SMZ800N, Nikon, Japan). Spiders were identified using the Dyntaxa database (SLU Artdatabanken 2024) and the taxonomy of the Global Biodiversity Information Facility (GBIF; https://www.gbif.org/). Centipedes were identified using relevant publications (Bortolin et al. 2018; Lewis et al. 2005; Stoev et al. 2010). All specimens were identified to the lowest possible taxonomic level using relevant literature and identification keys, with identifications confirmed by taxonomic experts Huiqin Ma (Hengshui University) and Yejie Lin (Imperial College London). Selected specimens were also validated using COI barcoding (Hebert et al. 2003); the results will be reported in a forthcoming study. Individual centipedes and spiders were oven‐dried at 65°C and weighed for biomass determination.

### 2.4 Environmental Variables

To explore potential drivers of centipede and spider communities along the elevation gradient, we measured the following habitat‐related factors based on the litter samples taken per plot: total carbon (TC), total nitrogen (TN) (both measured using an elemental analyzer; vario MARCRO cube, Elementar, Hamburg, Germany) and total phosphorus (TP) (measured via H_2_SO_4_—HClO_4_ digestion) concentrations, as well as pH in an aqueous suspension (litter:water = 1:2.5, w/v) (using a pH meter; Thermo Fisher Scientific Inc., San Jose, CA, USA). From these measurements, we calculated the carbon‐to‐nitrogen (C/N), carbon‐to‐phosphorus (C/P), and nitrogen‐to‐phosphorus (N/P) ratios in litter. Based on the latitude and longitude coordinates of sampling plots, we downloaded climate data from WorldClim (https://www. worldclim.org/) to extract annual mean temperature and annual mean precipitation data.

### 2.5 Statistical Analysis

Data analyses were performed in R software (v. 4.4.0; R Core Team 2024). Individual‐based sample completeness curves for the eight elevations, along with 95% unconditioned confidence intervals, were constructed using the “iNEXT” package (Hsieh et al. 2016). Functions from the “vegan” package (Oksanen et al. 2015) were used for subsequent analysis. Species accumulation curves were generated using the “specaccum” function. For each plot, density (ind./m^2^) and richness of both centipedes and spiders were calculated. Shannon diversity (Hill number, *q* = 1) was calculated using the “hill_taxa” function in the “hillR” package to represent the effective number of species in each community. Principal coordinates analysis (PCoA) based on Bray‐Curtis dissimilarities was conducted to assess variations in centipede and spider community composition across the elevation gradient using the “pcoa” function. Additionally, differences in community composition were assessed using permutational multivariate analysis of variance (PERMANOVA) using the “adonis” function. Pairwise differences in the response variables between elevations were assessed using Mann–Whitney *U* tests as implemented in the “rstatix” package. In addition, the environmental factors influencing response variables were assessed using Spearman correlations as implemented in the “Hmisc” package. Redundancy analysis (RDA) was performed to inspect correlations between environmental factors and community composition using the forward selection model as implemented in the “ordistep” function. Monte Carlo tests with 999 permutations were performed to evaluate the overall model significance. Variation explained by the selected environmental variables was assessed by adjusted *R* ^2^ values. The scaling option was set as “species” and the first 15 species strongly correlating with the first two axes were displayed. The silhouette images of centipedes and spiders were freely obtained from PHYLOPIC (http://phylopic.org/).

## 3 Results

### 3.1 Density, Diversity and Biomass

A total of 2796 centipedes and 2327 spider individuals were sampled, representing 26 species from two families of centipedes and 76 species from 18 families of spiders. Species accumulation curves of centipedes and spiders became marginally asymptotic, indicating that most of the species present at our study sites had been sampled (Figure S1a,c). Sample completeness curves further indicated that the centipedes and spiders sampled adequately represented the respective communities across the studied elevation gradient (Figure S1b,d). Several centipede species, including *Lithobius* sp.1, sp.2, sp.3, *L*. (*Chinobius*)* opinatus*, *L*. (*Chinobius*) sp.6, and *Geophilus* sp.1, occurred at each of the elevations (Figure S7), while others were restricted to specific elevations, such as *Lithobius* sp.8 and sp.13 found only at 800 and 950 m, and *Lithobius* sp.19 and *L*. (*Chinobius*) *aberrans* limited to 1850 m. Spider richness peaked at 800 m with 52 species, including several species unique to this elevation, while the lowest richness occurred at 1550 m (23 species). Eleven spider species were widespread across most elevations, and seven species were exclusive to 1850 m (Figure S8).

**FIGURE 2:**
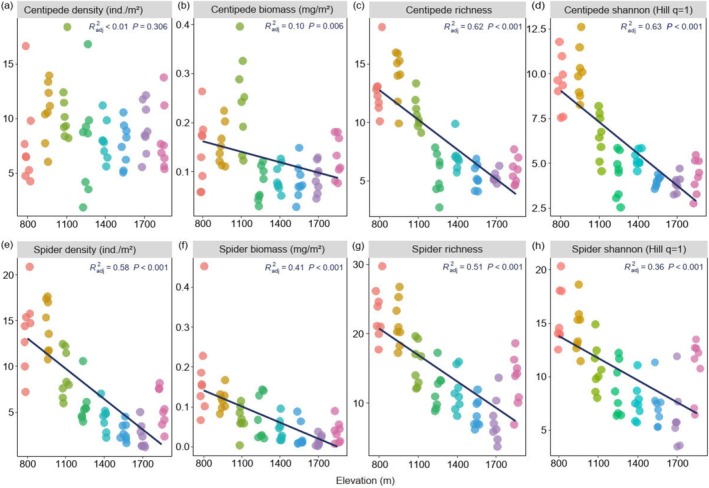
Changes in density (ind./m^2^), biomass (mg/m^2^), richness, Shannon (Hill number = 1) of centipedes and spiders along an elevation gradient on the Changbai Mountain, northeast China. The relationships are based on linear models and selected based on adjusted *R* ^2^, root mean square error, and Akaike information criterion; *p*‐values indicate significance levels.

Species richness (*F*_1,62_ = 103.06, *p* < 0.001; *F*_1,62_ = 69.20, *p* < 0.001), Shannon diversity (*F*_1,62_ = 106.69, *p* < 0.001; *F*_1,62_ = 36.26, *p* < 0.001) and biomass (*F*_1,62_ = 8.05, *p* = 0.006; *F*_1,62_ = 44.61, *p* < 0.001) of both centipedes and spiders, as well as spider density (*F*_1,62_ = 89.22, *p* < 0.001), significantly decreased with elevation (Figure 2). By contrast, centipede density (*F*_1,62_ = 1.07, *p* = 0.310) did not change significantly, and centipede biomass did not consistently decline with elevation but peaked at 1100 m. In spiders, but not in centipedes, the decline in biomass with elevation was less pronounced than that of density. At low elevations, spiders were more abundant than centipedes, but the opposite was true at high elevations. Generally, across elevations, the mean biomass of centipedes (12.5 ± 6.0 mg/m^2^) was 1.8 times higher than that of spiders (7.1 ± 4.0 mg/m^2^).

According to PCoA results, the community composition of centipedes (PERMANOVA, *R* ^2^ = 6.02, *p* < 0.001; Figure S2a) and spiders (*R* ^2^ = 6.63, *p* < 0.001; Figure S2b) varied significantly along the elevational gradient.

### 3.2 Drivers of Density, Diversity and Biomass

**FIGURE 3:**
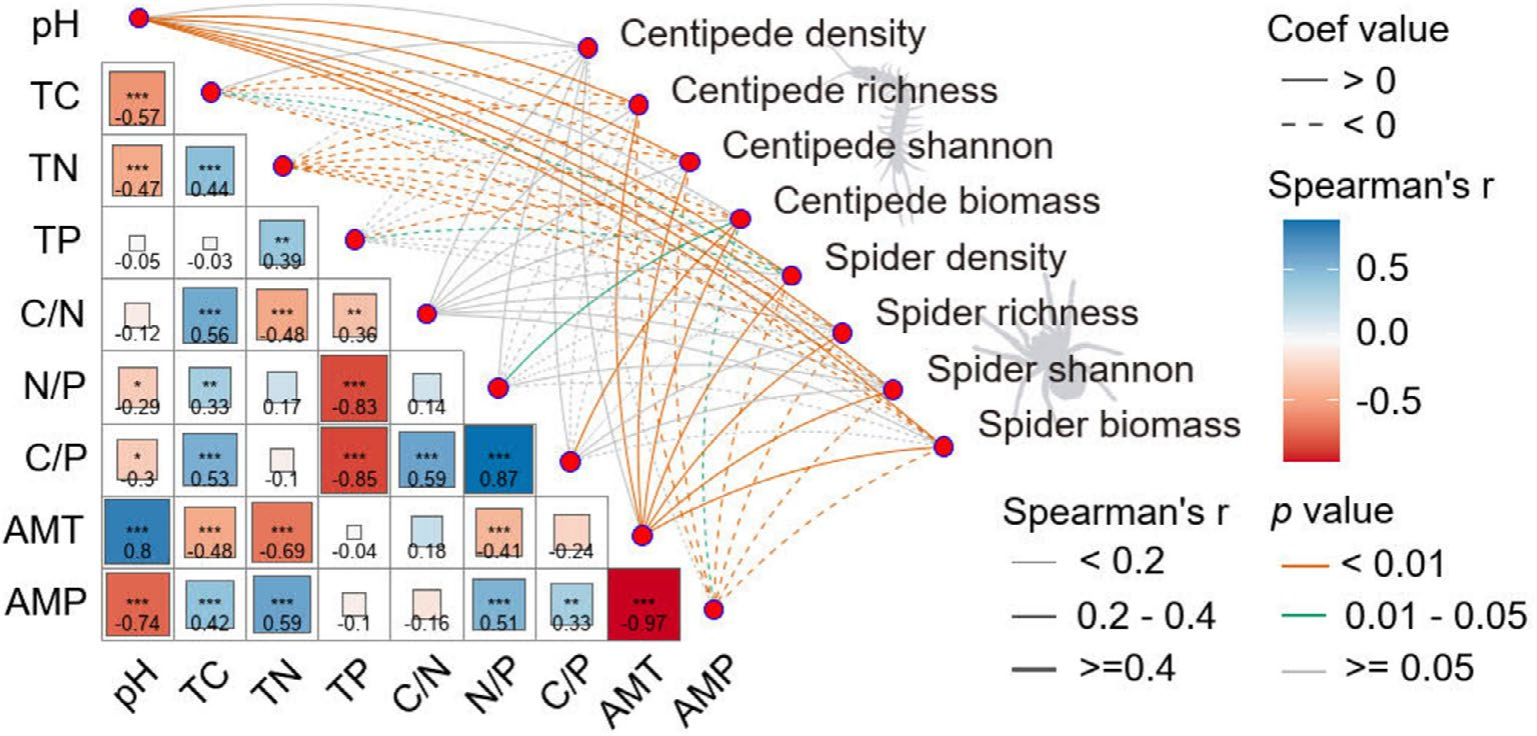
Spearman correlation coefficients (*r*; color coded) between the environmental factors studied, as well as between environmental factors (AMP, annual mean precipitation; AMT, annual mean temperature; C/N, litter carbon‐to‐nitrogen ratio; C/P, litter carbon‐to‐phosphorus ratio; pH, litter pH; N/P, litter nitrogen‐to‐phosphorus ratio; TC, litter total carbon; TN, litter total nitrogen; TP, litter total phosphorus) and density (ind./m^2^), richness, Shannon (Hill number = 1) and biomass (mg/m^2^) of centipedes and spiders; ***p < 0.001, **p < 0.01, *p < 0.05; line color indicates statistical significance, and dashed and solid lines indicate positive and negative correlation coefficients, respectively; line width indicates the strength of the correlation.

Spearman correlation analyses indicated that species richness and Shannon diversity of centipedes and spiders were positively correlated with annual mean temperature and litter pH and negatively correlated with annual mean precipitation and total nitrogen concentrations (Figures 3 and S3–, S6). Species richness and Shannon diversity of centipedes, as well as density of spiders, were negatively correlated with litter total carbon. Generally, spider community parameters more closely correlated with environmental factors than those of centipedes, with density significantly correlating with litter total phosphorus concentration.

The biomass of both centipedes and spiders was significantly positively correlated with annual mean temperature and negatively with annual mean precipitation and litter total nitrogen concentration (Figures 3 and S3–, S6). However, centipede biomass also correlated significantly with litter total phosphorus concentration and C/P ratio, while spider biomass also correlated with litter pH and total litter carbon concentration.

### 3.3 Drivers of Variations in Community Composition

**FIGURE 4:**
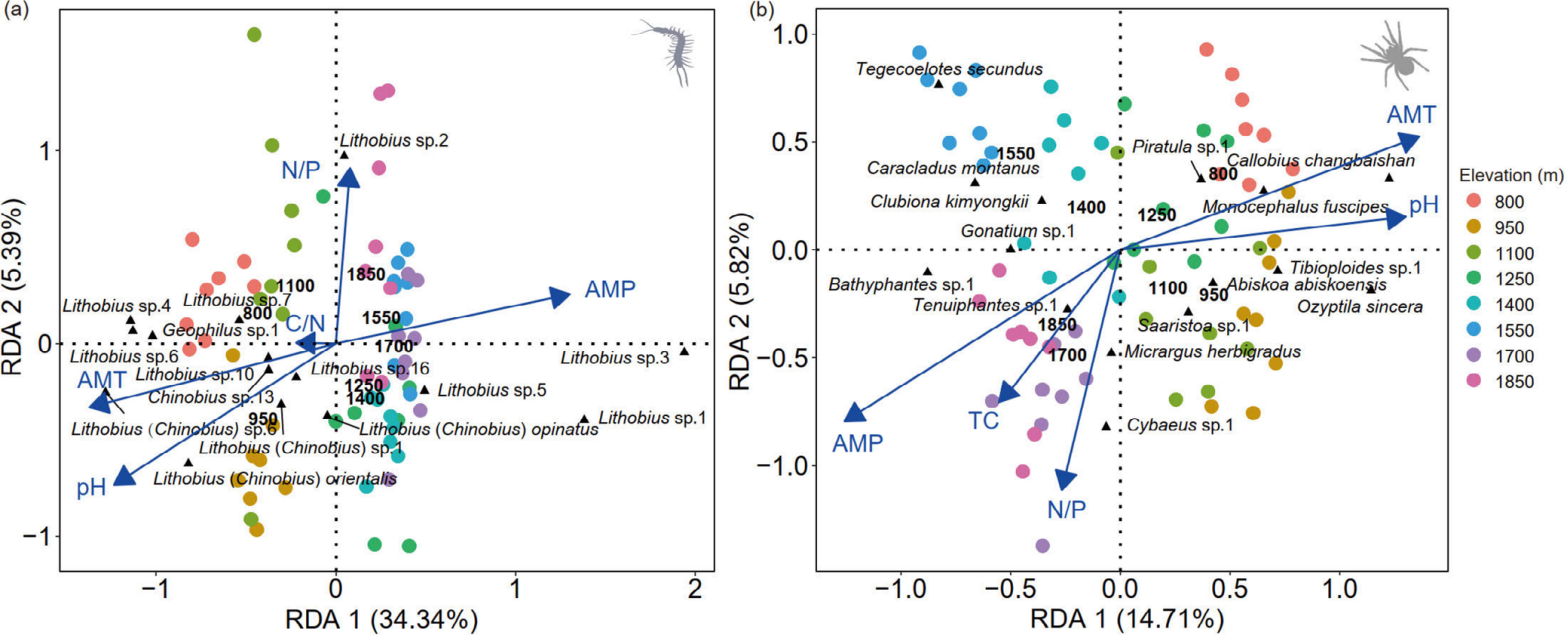
Redundancy analysis (RDA) on the relationship between centipede (a) and spider community composition (b) and environmental factors (AMP, annual mean precipitation (mm); AMT, annual mean temperature (°C); C/N, litter C/N ratio; N/P, litter N/P ratio, pH, litter pH; TC, litter total carbon) on Changbai Mountain. The length of arrows represents the percentage of variation explained by the respective variable. The 15 species most closely correlating with the first two axes are displayed.

Five factors were identified significantly affecting centipede and spider community composition along the studied elevation gradient by the forward selection procedure of the RDA (overall Monte Carlo test, *p* = 0.001 for both; Figure 4). These variables explained 39.7% (*F* = 6.02, *p* = 0.001) and 20.5% (*F* = 2.81, *p* = 0.001) of the variation in centipede and spider community composition, respectively. Mean annual temperature, mean annual precipitation, litter pH, and N/P ratios correlated with both centipede and spider community composition. In addition, the litter C/N ratio correlated with centipede community composition, and total litter carbon correlated with spider community composition.

Centipede communities at elevations between 1250 and 1850 m correlated closely with annual mean precipitation, with high densities of *Lithobius* sp.1, *Lithobius* sp.3, and *Lithobius* sp.5. Centipede communities at elevations between 800 and 1100 m correlated with annual mean temperature and litter pH, with high densities of *Lithobius* (*Chinobius*) sp.6, *Lithobius* (*Chinobius*) *orientalis*, *Geophilus* sp.1, and *Lithobius* sp.6. Spider communities at elevations between 800 and 1250 m correlated closely with litter pH and annual mean temperature, with high densities of *Callobius changbaishan*, *Monocephalus fuscipes* , *Ozyptila sincera* , and *Piratula* sp.1. Annual mean precipitation, litter total carbon, and N/P ratio correlated positively with spider communities at 1700 and 1850 m and were associated with high densities of *Micrargus herbigradus* and *Tenuiphantes* sp.1.

## 4 Discussion

### 4.1 Changes in Diversity and Biomass

Consistent with our Hypothesis 1, species richness and Shannon diversity of both centipedes and spiders, as well as spider density, decreased significantly with increasing elevation. The findings are in line with previous studies on canopy spiders (Wu et al. 2024) and soil oribatid mites (Liu et al. 2023a) on Changbai Mountain. Similar patterns have also been observed in soil animal taxa in other mountain regions, such as oribatid mites on Kinabalu Mountain in Borneo (Hasegawa et al. 2006) and on the Caucasus Mountains (Mumladze et al. 2017), as well as spiders in the Pico da Neblina Mountains in Brazil (Nogueira et al. 2021). They generally are consistent with the perspective that soil animals are sensitive to changes in environmental conditions, with changes in temperature being of major importance (Binkenstein et al. 2018; Blackburn et al. 2002). However, although the diversity of both taxa decreased with elevation, this was not the case for centipede density. This was mainly due to the increased density of the lithobiomorph species *Lithobius* sp.1 and *Lithobius* sp.3 at high elevations. The density of the species *Lithobius* (*Chinobius*) *opinatus* remained stable across the elevation gradient; the species *Lithobius* (*Chinobius*) *opinatus* is widespread in western Siberia and well adapted to cold winters (Nefediev and Farzalieva 2020) and thereby also well adapted to colonize high mountain regions. Interestingly, a similarly well adapted species of spiders reaching high density and biomass at high elevations appears to be lacking.

Similar to species richness and diversity, the biomass of centipedes and spiders decreased significantly with increasing elevation, further supporting our Hypothesis 1. These patterns, however, were not consistent with the results of an earlier study on the biomass of litter invertebrates on Dongling Mountain, Beijing, China, which showed a hump‐shaped relationship with elevation peaking at 1400 m (Xu et al. 2017). Similar to these findings, the biomass and density of centipedes on Changbai Mountain did not uniformly decline with elevation but peaked at 1100 m. Presumably, this is related to high prey availability; as on Changbai Mountain, the density of Collembola, as a major prey of centipedes, also peaks at 1100 m (Xie, Sun, et al. 2022). In addition, sites at 1100 m represent the transition zone between broad‐leaved mixed forest and mixed coniferous forest and support high vegetation diversity (Sang and Bai 2009), which may contribute to the high density of centipedes and Collembola. By contrast, spider diversity and biomass peaked at lower elevations (800–950 m), possibly due to higher primary productivity and a more complex vegetation structure known to favor spider communities (Birkhofer and Wolters 2012; Ramos et al. 2022; Wang et al. 2006).

On average, centipede biomass exceeded that of spiders by a factor of 1.8, likely related to the larger body size of the former (Albert 1983; Bartos 2005). Further, their soil‐dwelling behavior allows centipedes to retreat deeper into the soil, thereby avoiding harsh climatic conditions at the soil surface, promoting population stability (Hembree 2023). By contrast, spiders—particularly web‐building species—rely on aboveground vegetation (Malumbres‐Olarte et al. 2013), making them more vulnerable to environmental harshness and seasonal changes in vegetation and habitat structure. These differences highlight that life history traits and habitat preferences need to be considered for understanding elevation patterns in soil invertebrates.

### 4.2 Driving Factors of Centipede and Spider Diversity, Biomass and Community Composition

Supporting our Hypothesis 2, temperature and precipitation were identified as the major drivers of species richness, biomass, and community composition in both centipedes and spiders, as well as spider density. Specifically, richness and biomass were positively correlated with temperature and negatively correlated with precipitation, aligning with previous findings (Castillo‐Avila et al. 2025).

As poikilotherm animals, centipedes and spiders are highly sensitive to temperature, which affects their metabolism (Block 1981; Gillooly et al. 2001), reproduction (Kristiansen et al. 2024; Malzahn et al. 2003) and geographic distribution (Shoutmaus 2023). Warmer environments extend the activity periods of centipedes and accelerate egg development (Adis et al. 1996; de Oliveira et al. 2019; Lewis 2007). For example, tropical centipedes may stay active throughout the year, whereas temperate species are only active for4–6 months in spring and summer (Lewis 2007). Longer activity periods allow for more generations and prolonged foraging, contributing to higher biomass (Hirakizawa and Yamauchi 2021). Similarly, elevated temperatures enhance spider performance by increasing web‐building efficiency, silk flexibility, and predation success (Barghusen et al. 1997; DeLong et al. 2023; Jiang and Nayeb‐Hashemi 2020).

In contrast, high precipitation negatively affected the diversity and biomass of centipedes and spiders. Excess rainfall can block soil pore spaces used by centipedes (Ivask et al. 2019), collapse their habitat structure (Baalbergen and Donovan 2013), and increase predation risk by forcing surface exposure (Castillo‐Figueroa and Castillo‐Avila 2025a, 2025b). In spiders, especially web‐builders, heavy rainfall can damage webs, leading to increased energetic costs for repair and relocation (Almeida and Gasnier 2017; Blamires and Sellers 2019; Majer et al. 2015; Yazawa et al. 2020). Thus, precipitation imposes direct and indirect constraints on predator communities.

In addition to broad‐scale climatic variables, litter chemistry also significantly influenced centipede and spider diversity, biomass, and community composition—supporting Hypothesis 2. While litter pH and total nitrogen (TN) impacted both taxa, litter C/N and C/P ratios had stronger effects on centipedes. Soil pH is a known determinant of microbial and invertebrate diversity, with optimal communities forming under moderately acidic to neutral conditions (Fierer and Jackson 2006; Johnston 2019; Johnston and Sibly 2020). High litter nutrient concentrations (TC, TN, TP) were negatively correlated with richness, diversity, and biomass of both groups. Among the nutrients, calcium has been shown to play a particularly important role in shaping soil invertebrate communities, influencing both abundance and composition (Castillo‐Avila et al. 2025; Mamabolo et al. 2024; Ohta et al. 2014). These findings suggest that centipedes and spiders benefit from low‐quality, slow‐decomposing litter that accumulates on the soil surface (Castillo‐Figueroa and Castillo‐Avila 2025a, 2025b).

Low‐nutrient litter likely contributes to greater habitat complexity, which benefits predator communities by providing refuge and increasing prey availability (Castillo‐Figueroa and Posada 2025; Finke and Denno 2006; Schmidt and Rypstra 2010). Thicker litter layers also support detritivore populations, such as Collembola, that form key prey for centipedes and spiders (Agustí et al. 2003; Eaton 2006; Pollierer and Scheu 2017; Saitoh et al. 2011). The stronger litter effect observed in spiders suggests they may rely more heavily on surface litter habitats and associated prey, while centipedes may forage more in soil pore spaces.

The elevation‐driven shifts in species composition and biomass further underscore the role of temperature and habitat structure. At low elevations (800–1100 m), larger‐bodied centipede species (e.g., *Lithobius* (*Chinobius*) sp.6, *L. orientalis* , *Geophilus* sp.1) and spiders (e.g., *O. sincera* , *Piratula* sp.1, *C. changbaishan*) were dominant, whereas smaller‐bodied species (e.g., *Lithobius* sp.1, *M. herbigradus* , *Tenuiphantes* sp.1) increased in relative abundance at mid‐to‐high elevations (1250–1850 m). These patterns suggest that lower temperatures and shorter growing seasons at higher elevations may constrain growth and reproduction in larger‐bodied predators.

Together, our results highlight the interactive roles of climatic variables and litter chemistry in structuring soil predator communities. Temperature and precipitation primarily drive large‐scale patterns in diversity and biomass, while litter characteristics mediate community composition through bottom‐up effects and habitat complexity. The effects of litter nutrient concentrations were more pronounced in spiders than in centipedes, possibly because spiders rely more on prey associated with accumulated surface litter, whereas centipedes forage more extensively in the pore spaces of mineral soil. These findings emphasize the need to consider both broad‐scale climatic factors and local habitat conditions when assessing soil biodiversity along elevational gradients.

## 5 Conclusions

We examined the effects of climatic variables and litter characteristics on centipede and spider density, diversity, biomass, and community composition in forests across an elevation gradient. Centipede and spider species richness, Shannon diversity, and biomass, as well as spider density, decreased significantly with elevation, primarily driven by annual mean temperature and precipitation. The reduction in predator diversity at higher elevations could alter the regulation of detritivore populations and ultimately affect decomposition dynamics. Additionally, litter nutrient concentrations also shaped community patterns, with a negative effect on density, diversity, and biomass, suggesting that both taxa benefit from slowly decomposing litter. Overall, the findings highlight the importance of both broad‐scale climatic drivers and local litter conditions in structuring soil predator communities. The close associations observed between environmental variables and community attributes suggest that deterministic processes, such as environmental filtering, play a central role in structuring these communities along elevational gradients. Future studies incorporating trait‐based or phylogenetic analyses are encouraged to further investigate these assembly mechanisms. Given the ecological roles of centipedes and spiders as key predators in soil food webs, changes in their diversity and biomass may affect litter decomposition, nutrient cycling, and ecosystem stability. Our study was based on a single‐time sampling effort conducted on one mountain, and the lack of soil physicochemical data may limit our understanding of additional drivers shaping community structure. To address these limitations, future research should include repeated samplings across seasons and regions, integrate detailed soil property measurements, and apply functional or phylogenetic frameworks to better understand the drivers of soil predator communities and inform conservation strategies aimed at preserving their diversity and ecological function.

## Author Contributions

**Zhuoma Wan:** data curation (equal), formal analysis (equal), software (equal), validation (equal), visualization (equal), writing – original draft (equal). **Yunga Wu:** data curation (equal), investigation (equal), writing – review and editing (equal). **Peng Zhang:** formal analysis (equal), writing – review and editing (equal). **Zhijing Xie:** formal analysis (equal), software (equal), writing – review and editing (equal). **Donghui Wu:** conceptualization (equal), supervision (equal), writing – review and editing (equal). **Stefan Scheu:** conceptualization (equal), supervision (equal), writing – review and editing (equal).

## Conflicts of Interest

The authors declare no conflicts of interest.

## Supporting information


**Figure S1:** ece372074‐sup‐0001‐FigureS1.jpg.

**Figure S2:** ece372074‐sup‐0002‐FigureS2.jpg.

**Figure S3:** ece372074‐sup‐0003‐FigureS3.jpg.

**Figure S4:** ece372074‐sup‐0004‐FigureS4.jpg.

**Figure S5:** ece372074‐sup‐0005‐FigureS5.jpg.

**Figure S6:** ece372074‐sup‐0006‐FigureS6.jpg.

**Figure S7:** ece372074‐sup‐0007‐FigureS7.jpg.

**Figure S8:** ece372074‐sup‐0008‐FigureS8.jpg.

## Data Availability

Data associated with this study are available from the Dryad Digital Repository: https://doi.org/10.5061/dryad.866t1g22n.
