# Supplementary figures and images for: Drivers of Centipede and Spider Diversity and Biomass Along an Elevation Gradient on Changbai Mountain, China
Source: Ecol Evol. 2025 Sep 25;15(10):e72074. doi: 10.1002/ece3.72074 (PMC12461124; doi:10.1002/ece3.72074)

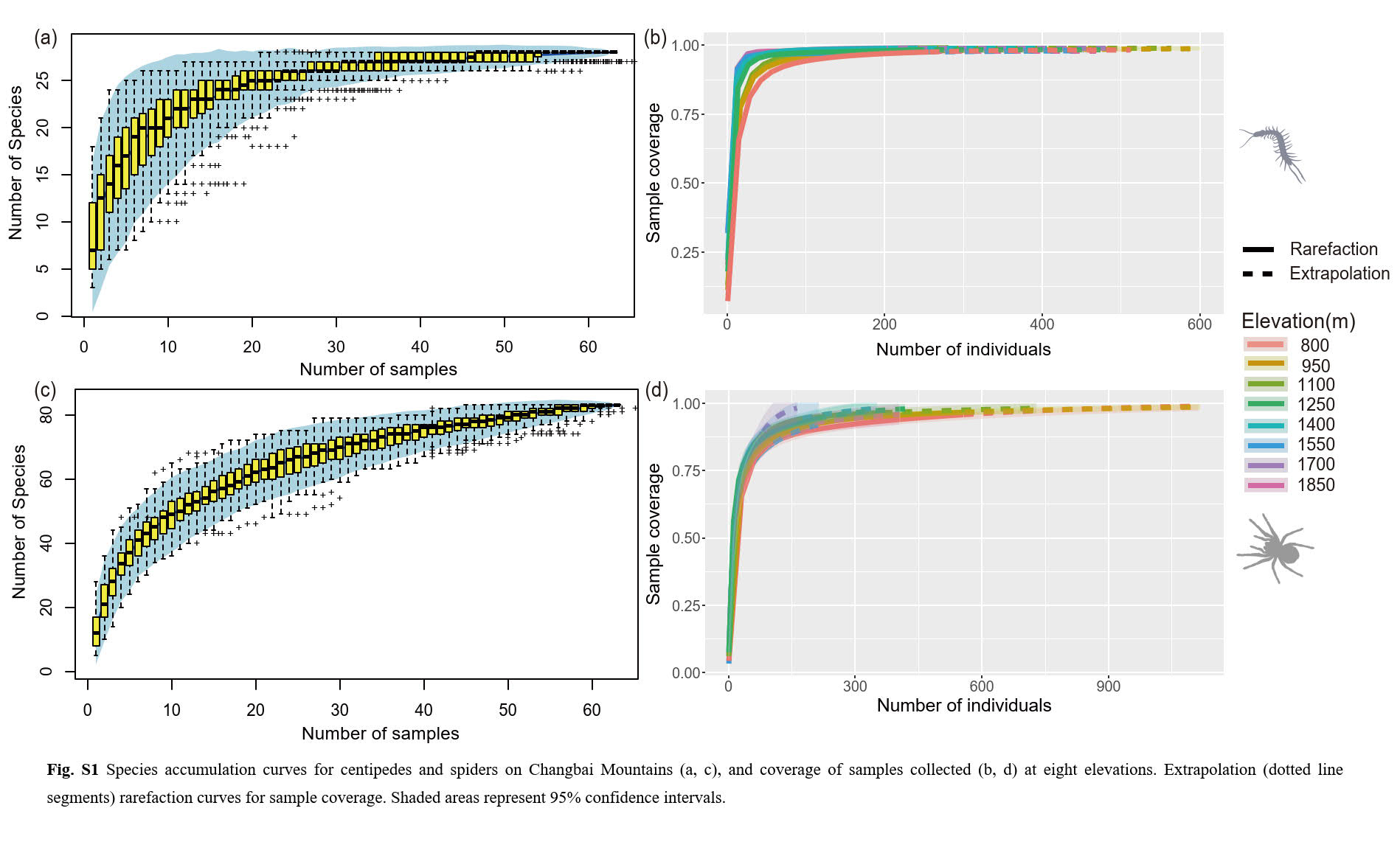

Supplement: Supplementary file 1 — Figure S1: ece372074‐sup‐0001‐FigureS1.jpg. [file ECE3-15-e72074-s005.jpg]

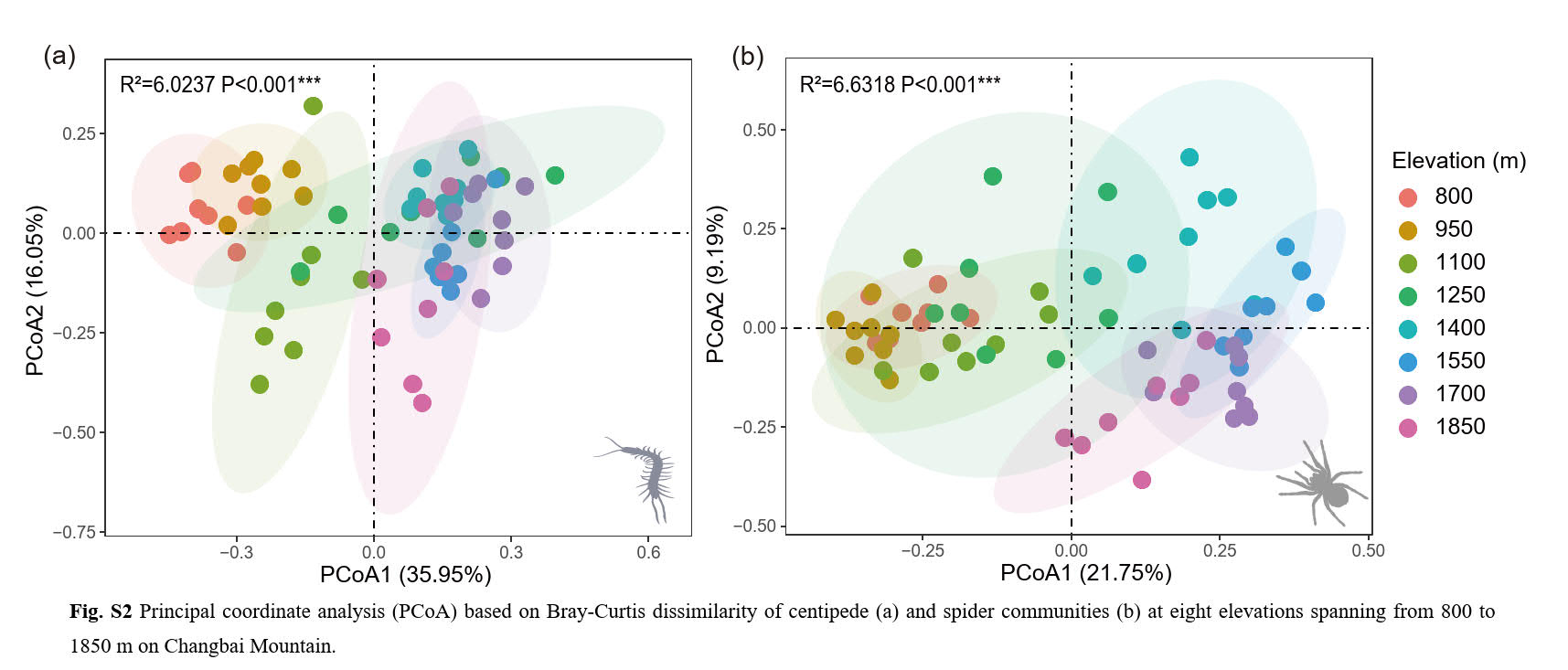

Supplement: Supplementary file 2 — Figure S2: ece372074‐sup‐0002‐FigureS2.jpg. [file ECE3-15-e72074-s007.jpg]

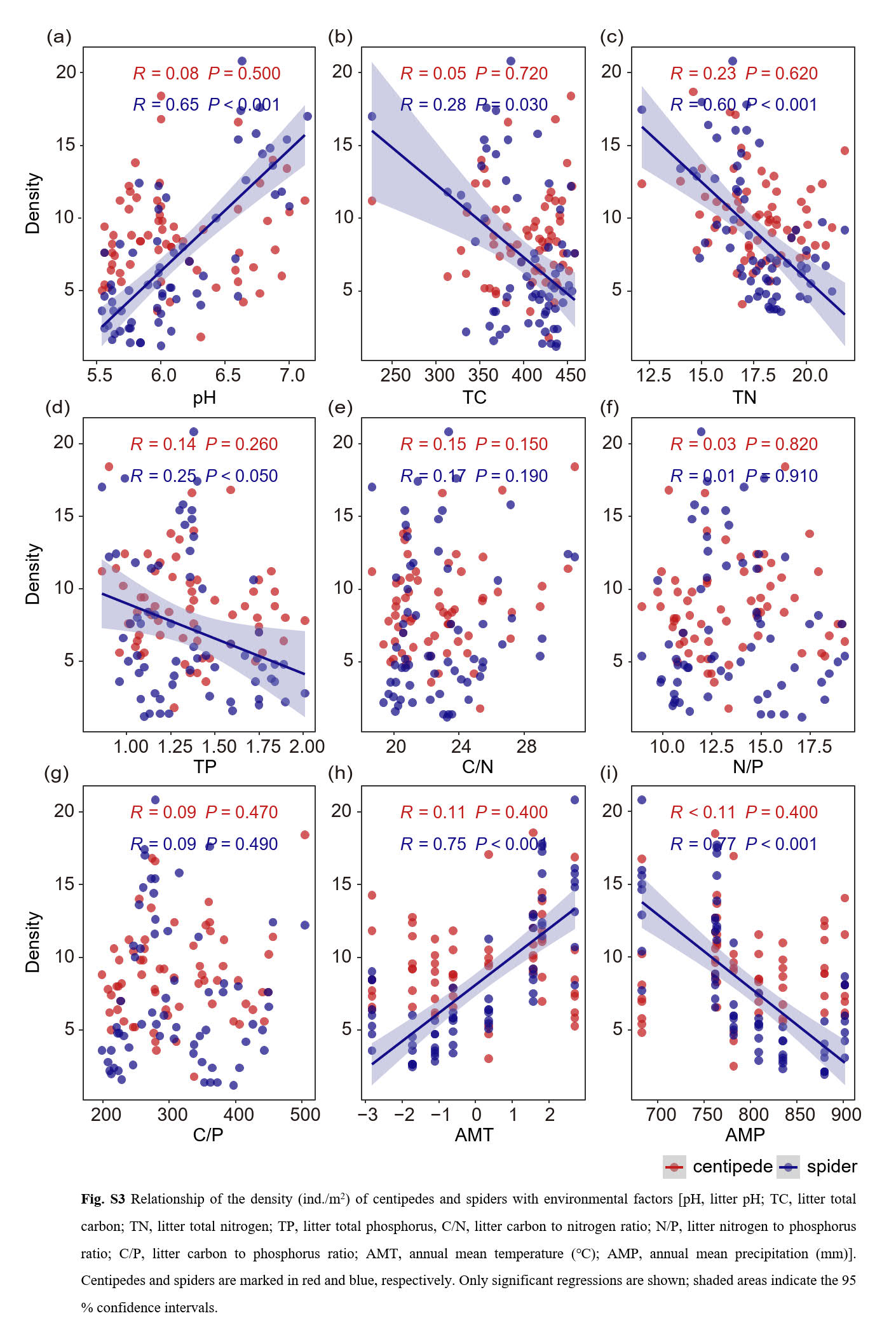

Supplement: Supplementary file 3 — Figure S3: ece372074‐sup‐0003‐FigureS3.jpg. [file ECE3-15-e72074-s002.jpg]

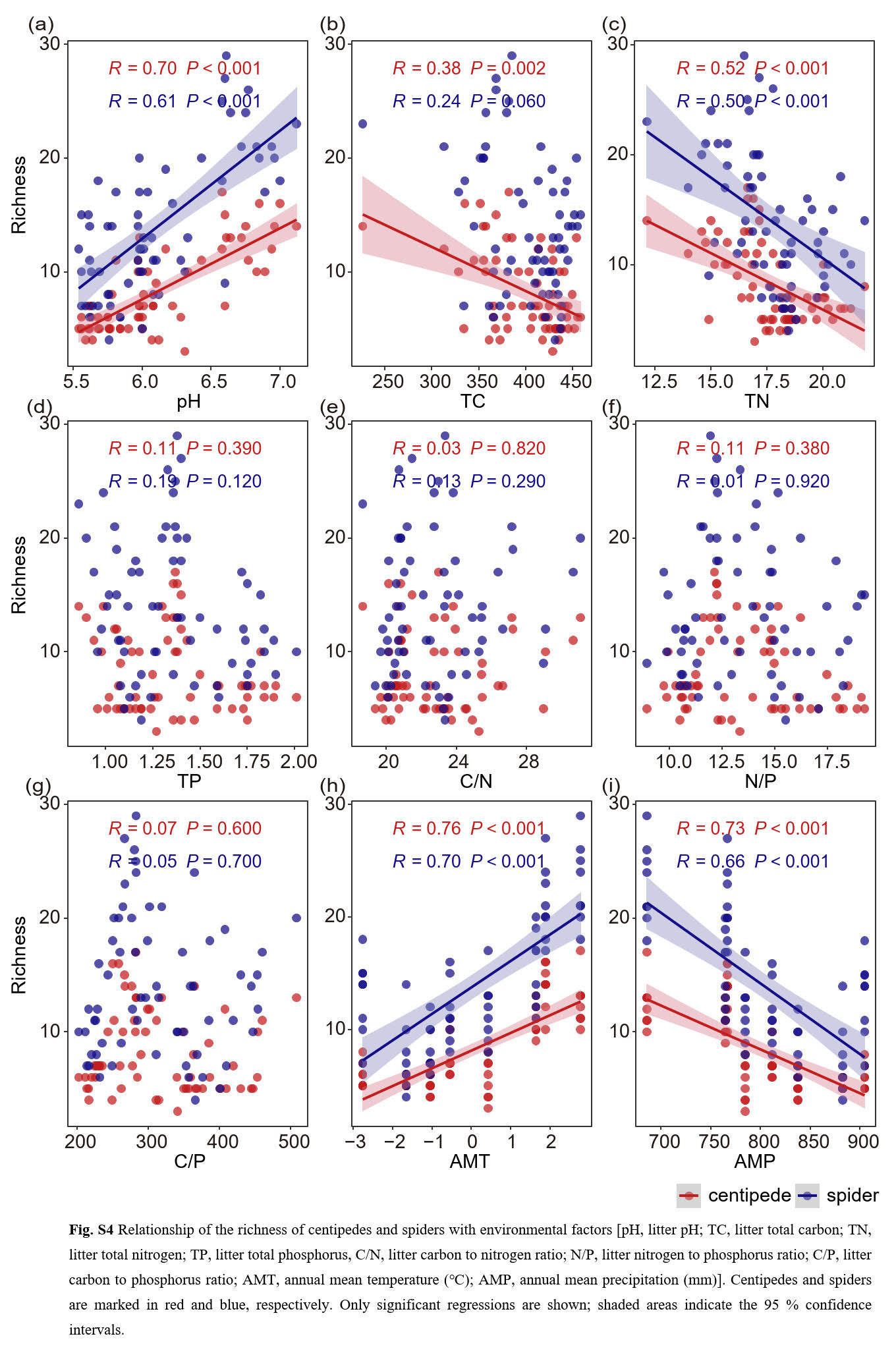

Supplement: Supplementary file 4 — Figure S4: ece372074‐sup‐0004‐FigureS4.jpg. [file ECE3-15-e72074-s008.jpg]

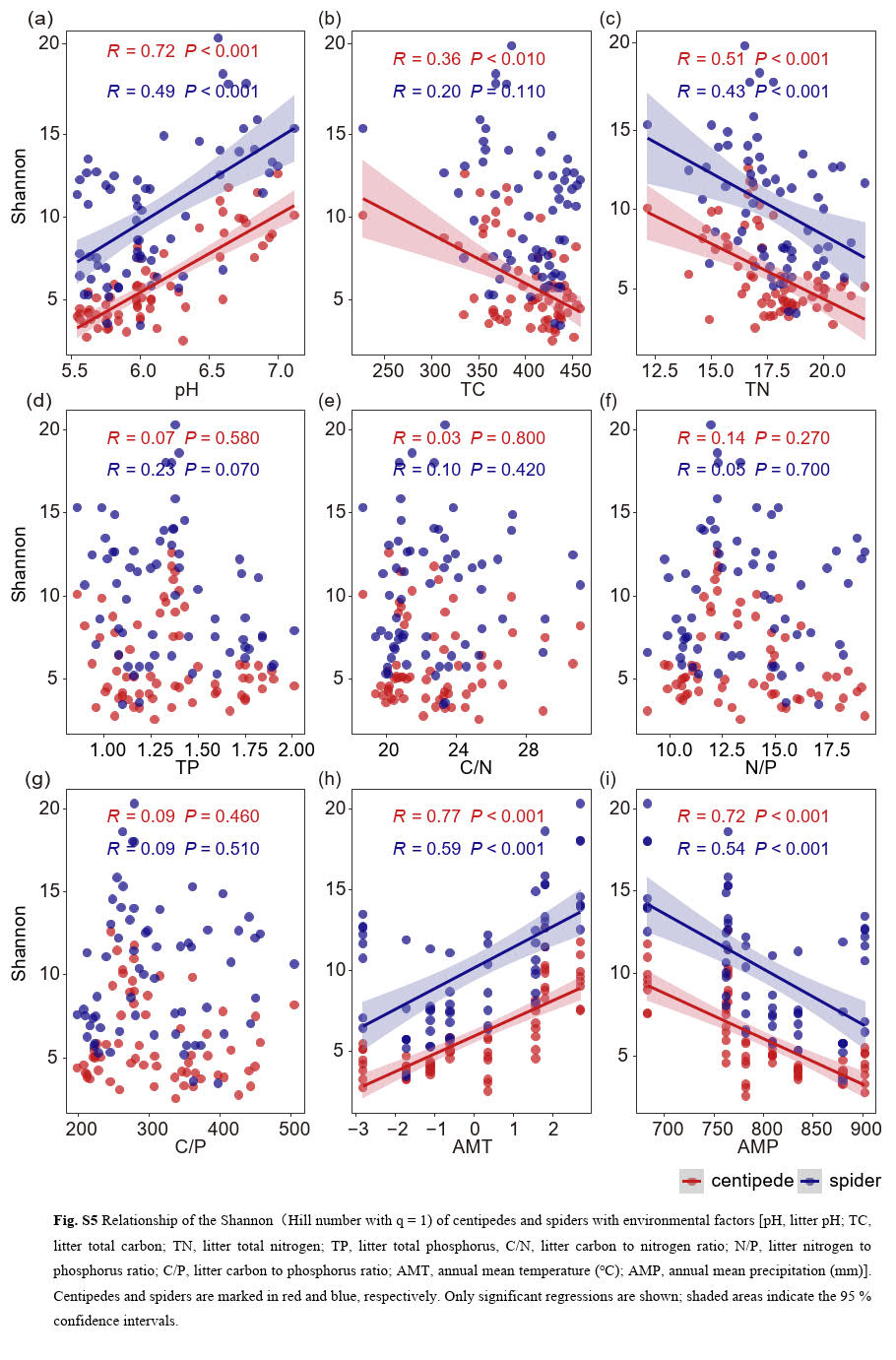

Supplement: Supplementary file 5 — Figure S5: ece372074‐sup‐0005‐FigureS5.jpg. [file ECE3-15-e72074-s006.jpg]

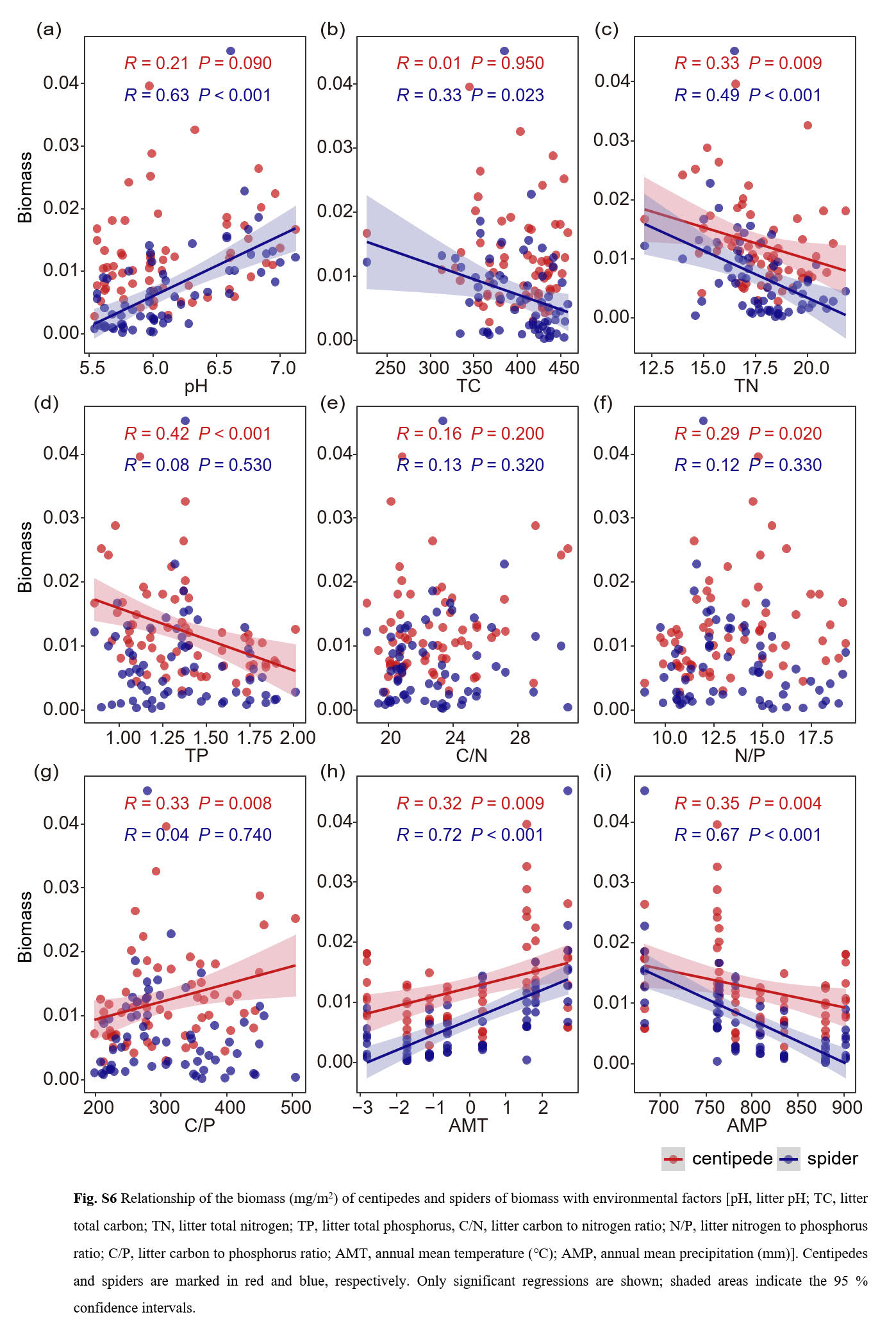

Supplement: Supplementary file 6 — Figure S6: ece372074‐sup‐0006‐FigureS6.jpg. [file ECE3-15-e72074-s004.jpg]

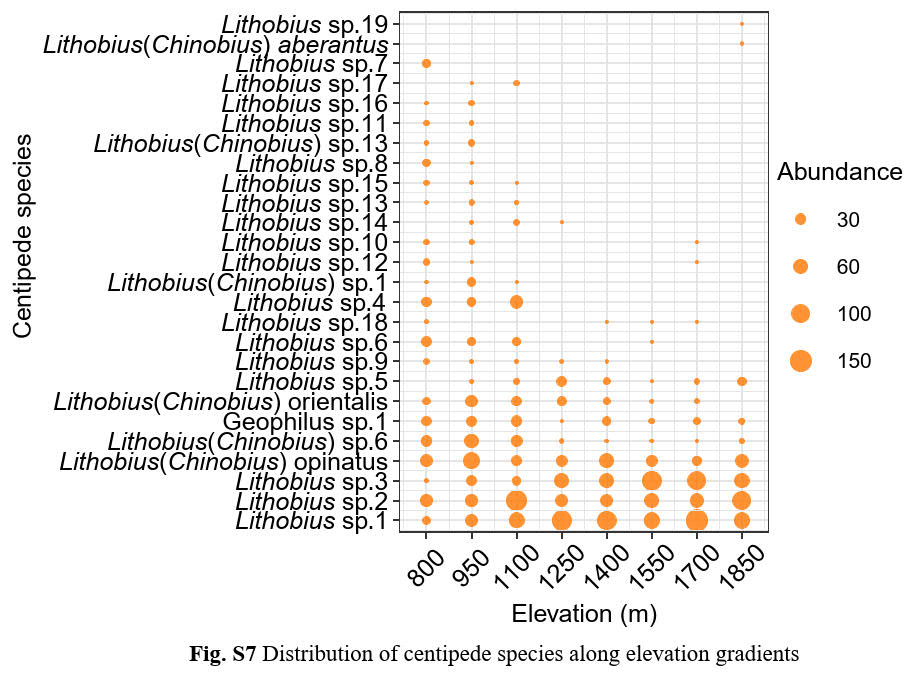

Supplement: Supplementary file 7 — Figure S7: ece372074‐sup‐0007‐FigureS7.jpg. [file ECE3-15-e72074-s003.jpg]

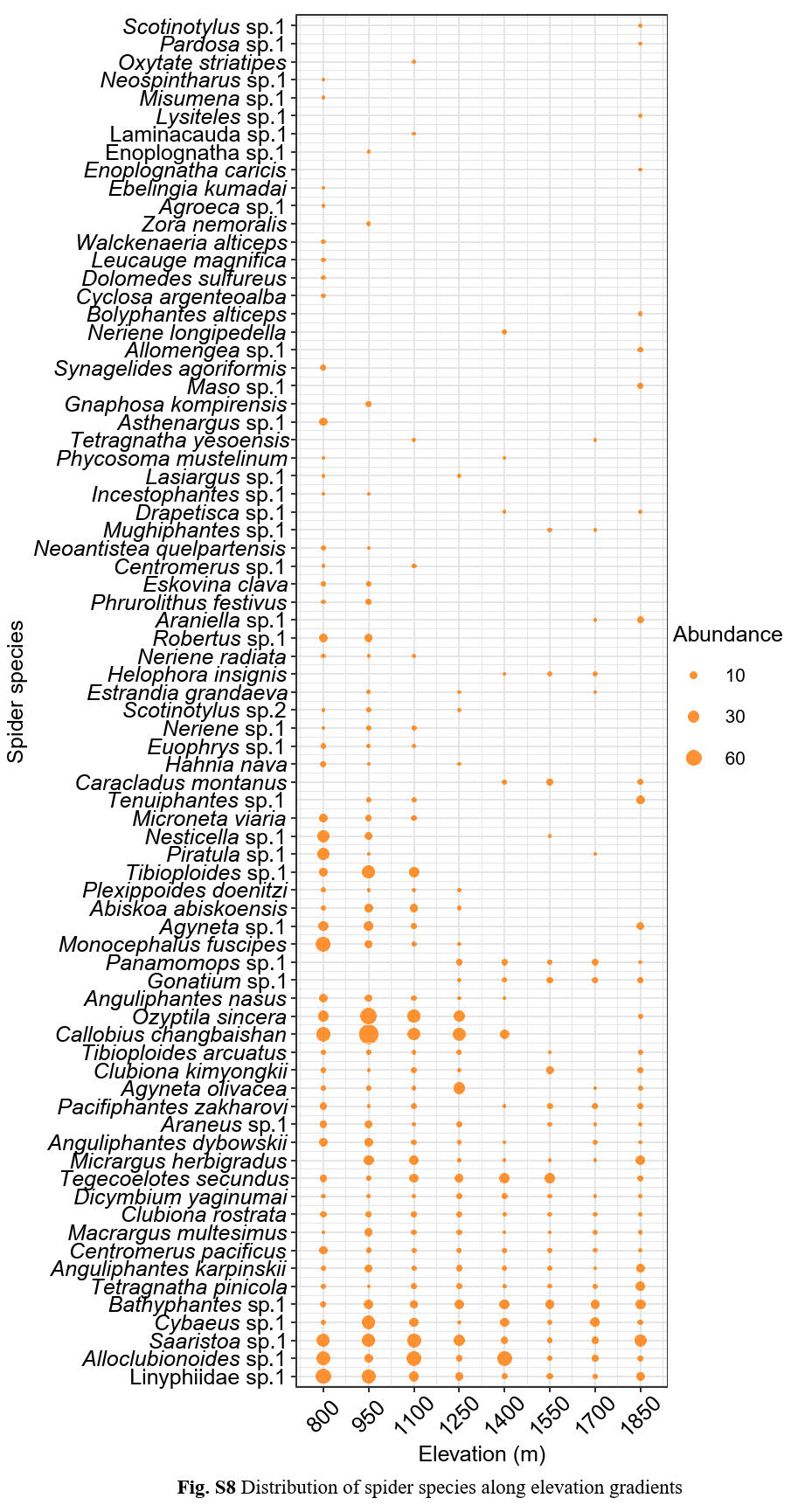

Supplement: Supplementary file 8 — Figure S8: ece372074‐sup‐0008‐FigureS8.jpg. [file ECE3-15-e72074-s001.jpg]
